# Evaluation of Precision Feeding to Enhance Broiler Growth Performance

**DOI:** 10.3390/ani15162433

**Published:** 2025-08-19

**Authors:** Aamir Nawab, Thi Hiep Dao, Peter V. Chrystal, David Cadogan, Stuart Wilkinson, Eunjoo Kim, Tamsyn Crowley, Reza Barekatain, Amy F. Moss

**Affiliations:** 1 School of Environmental and Rural Science, Faculty of Science, Agriculture, Business and Law, University of New England, Armidale, NSW 2351, Australia; anawab@myune.edu.au (A.N.); tdao2@une.edu.au (T.H.D.); eunjoo.kim@sydney.edu.au (E.K.); 2 Complete Feed Solutions, Hornsby, NSW 2077, Australia; peter@completefeeds.co.nz; 3 Feedworks Pty Ltd., Romsey, VIC 3434, Australia; david.cadogan@feedworks.com.au (D.C.);; 4 Poultry Hub Australia, University of New England, Armidale, NSW 2350, Australia; tamsyn.crowley@une.edu.au; 5 South Australian Research and Development Institute (SARDI), Roseworthy Campus, Roseworthy, SA 5371, Australia; reza.barekatain@sa.gov.au

**Keywords:** broiler, efficiency, feed cost, nutrient utilization, precision feeding

## Abstract

The effects of precision feeding regimes on broiler performance, organ weight, nutrient utilization, carcass yield, and calculated wholesale returns were investigated over 42 days. The treatments consisted of a standard four-phase commercial diet as the control, a precision nutrition blend diet based on a daily nutrient requirement, a precision nutrition adjusted diet based on weekly bird weight, and a standard commercial blend diet. Each dietary treatment was replicated 10 times with 11 birds per replicate. A total of 440 male Ross 308 (Aviagen, Goulburn, NSW, Australia) broiler chickens were offered experimental diets from days 11 to 42 post-hatch. Dietary treatments did not affect the feed intake and weight gain over the entire study. However, a reduced weight corrected FCR (higher feed efficiency) was observed in birds fed a precision nutrition adjusted blend diet compared to those fed the control diet from days 11 to 42 (*p* < 0.001). There were no significant differences in feed costs between treatments. Birds offered the precision nutrition adjusted diet improved AME (*p* = 0.002) measured from days 25 to 27 compared to the blended standard diet. Over the majority of time points, the precision nutrition adjusted diet significantly reduced the coefficient of variation in bird weight as compared to the control diet (*p* < 0.026).

## Simple Summary

Precision nutrition aims to meet birds’ nutrient requirements on a daily basis. An increase in feeding phases improves efficiency; however, the process of pelleting, transporting, and storing four or more separate diets is often impractical. Therefore, this study developed a precision nutrition program utilizing only two dietary concentrates blended on a daily basis to meet the daily energy and protein requirements of broilers using modern feeding technology. Precision nutrition treatments significantly improved the efficiency of chicken-meat production.

## 1. Introduction

Poultry is the most consumed animal protein source in the world, and the trend is expected to continue increasing [1]. The world population reached 8 billion in 2022, and is likely to reach 9 billion in 2037 [2], which will continue to increase pressure on the global chicken-meat industry. One way to meet this increasing demand is to improve the efficiency of chicken-meat production. Precision nutrition is a strategy to improve growth efficiency, reducing the resources needed for chicken-meat production and improving the profitability of the chicken-meat industry. The concept of precision nutrition aims to meet the birds’ daily nutrient requirement according to their growth rate. Broiler production involves constant changes in nutrient requirements as they grow rapidly, so birds may not receive sufficient nutrients if they are under-fed throughout the production cycle [3]. Consequently, a precision nutrition regime can reduce nutrient waste by adjusting the nutrient supply to more closely meet the daily requirement and prevent over-feeding of nutrient [4]. It was reported that by implementing a precision nutrition regime, the feed efficiency of broilers increased by 4.6% [5]. Consequently, based on this figure, the Australian poultry industry could save AUD 60 million by reducing feed production by 145,000 tons, compared with birds on a standard program of four diet phases [5].

Kleyn [3] indicated that blending a precise ration to meet a broiler’s daily energy and lysine requirements improved feed efficiency and reduced the coefficient of variation, as compared to a two-phase diet program. Similar results were found in pigs, where precision feeding reduced apparent digestible lysine intake by 26% without compromising growth performance compared to conventional feeding, saving USD 7.60 per pig [6]. In addition to reduced production cost, a similar study in pigs showed that a precision nutrition program reduced nitrogen and phosphorus excretion by 38% compared to traditional phase feeding, potentially benefiting the environment [7]. It is thought that by using synthetic amino acids, precision nutrition ensures the right balance of apparent digestible amino acids (e.g., lysine, methionine, threonine), reducing excess crude protein and, in turn, contributing to less nitrogen waste in manure.

Many studies have suggested that increasing the number of feeding phases makes feeding more efficient; however, pelleting, transporting, and storing four or more separate diets is often impractical [5,8]. Nevertheless, modern feed blending systems can automatically blend dietary components daily to achieve the desired nutrient profile. Thus, a protein dense concentrate diet can be formulated for day-old chicks, which can then be diluted by an energy dense concentrate on a daily basis to meet the nutritional needs. As this process requires only two concentrates to meet the daily needs of broilers, this program may now not be limited by the practicalities of feed transportation and storage of multiple diets.

Despite these potential benefits, there have not been many studies performed on precision nutrition strategies for broilers, as precision nutrition requires the use of equipment that is not at present commonly used in broiler sheds due to historically prohibitive cost; including feed blending and delivery systems, plus the cost of the extra silos required (two per shed vs. one per shed). Further, advanced tools including near-infrared spectroscopy (NIRS), are also needed to ensure the accuracy of feed formulations, but may not be affordable for non-integrated producers. Nevertheless, with the cost of technology reducing over time, this equipment is rapidly becoming more affordable for commercial use. Thus, it is timely to revisit the potential of precision nutrition regimes to improve the efficiency of chicken-meat production. To do so, as most of the current information is from studies in pigs, further study in broiler chickens is needed to confirm if precision nutrition may be an effective strategy to improve the efficiency of production.

Therefore, this study explored the development and implementation of a precision feeding program that blends two dietary components to meet daily broiler energy and protein requirements via modern feeding technology, to test the hypothesis that precision nutrition will increase the efficiency and profitability of chicken-meat production. For the first time, this includes not only blending the diets but also adjusting the diet blends fed to birds based on their bodyweight to more accurately meet their requirements.

## 2. Materials and Methods

### 2.1. Experimental Design, Diets, and Housing

The study was approved by the University of New England’s Animal Ethics Committee (AEC20-106) and met the requirements of the Australian Code of Practice for the care and use of animals for scientific purposes [9].

Upon arrival, day-old Ross 308 male broiler chicks from the female parent line were fed a common starter diet for the first 10 days in a temperature-controlled room. An initial room temperature of 32 ± 1 °C was maintained for the first week, which was gradually decreased to 21 ± 1 °C by the end of the third week, and maintained at this temperature until the end of the study. On day 11, birds were weighed and evenly allocated to dietary treatments based on body weight with 10 replicates of 11 birds per treatment (11 birds/0.96 m^2^). Birds were raised in floor pens (120 cm × 80 cm), with wood shavings as bedding material (depth = 7 cm) in a temperature-controlled room from days 11 to 42 post-hatch. A hanging feeder and three water nipples were provided to birds in each pen during the study. Birds had free access to water and feed throughout the study. The treatments consisted of (Table 1 and Table 2) (1) a standard four-phase commercial diet as a control (starter, grower, finisher and withdrawal diets); (2) a precision nutrition blend diet based on a protein “Hi Pro” and energy “Lo Pro” concentrate blended to meet the calculated daily nutrient requirement; and (3) a precision nutrition adjusted diet, based on protein “Hi Pro” and energy “Lo Pro” concentrates blended to meet the calculated daily nutrient requirement as per the precision nutrition treatment (treatment 2). Additionally, the birds offered this treatment also had their blend proportions of the protein and energy concentrates adjusted weekly based on the actual weekly bird weight data (as opposed to treatment 2, which was based on the predicted data only). (4) The fourth treatment consisted of a blended standard diet where the 4-phase commercial diet was gradually blended so that after the start of each phase, the diet was gradually replaced with the next dietary phase to avoid sudden nutrient changes. Bird weight was measured weekly as the precision nutrition adjusted treatment required this frequency to adjust the feeding schedule accordingly. Two basal diets were formulated for precision nutrition blend diets, which comprised a high-protein and low-energy concentrate, and a low-protein and high-energy concentrate. These concentrates were formulated such that the high-protein blend contained sufficient protein (i.e., standardized ileal digestible lysine) to meet the highest protein requirement during the trial (i.e., the first day) while also having a low enough energy content to meet the energy requirement at that stage. Likewise, the energy concentrate was formulated to meet the highest energy requirement over the trial (i.e., the last day) while having a low enough protein content to also meet the protein requirement at that stage. The remaining nutrients were formulated according to the breed (Ross 308 Broiler) nutrient requirements. Nutrient content of the dietary components, the daily blends offered, the calculated standardized ileal digestible lysine intake and AME intake based on the average intake of treatments is given in Table 3, Table 4 and Table 5, respectively. In the precision nutrition treatments, feed was provided ad libitum by giving the estimated daily feed intake (which was an accurate prediction, given we were measuring daily intake data) plus 10%. Any uneaten feed was weighed out on a daily basis if it was greater than approximately 200 g, immediately prior to the new blend being added. All the diets were based on wheat and soybean meal. Titanium dioxide (TiO_2_) was added in the withdrawal phase at 0.5% as an inert marker for digestibility analysis. Diets were mixed with a horizontal ribbon mixer for 15 min total (adding oil at 5 min duration). Diets were pelleted through a Palmer PP300 S/WIDE pellet press (Palmer Milling Engineering, Griffith, NSW, Australia) with a die diameter of 4.0 mm and pellet length of 0.5 mm. A ‘23 h on 1 h off’ lighting regime was applied for the first 3 days, followed by ‘20 h on 4 h off’ for 7 days and, finally, ‘18 h on 6 h off’ was used for the remainder of the study.

The composition and nutrient content of experimental diets are shown in Table 3 and Table 4.

**Figure 1:**
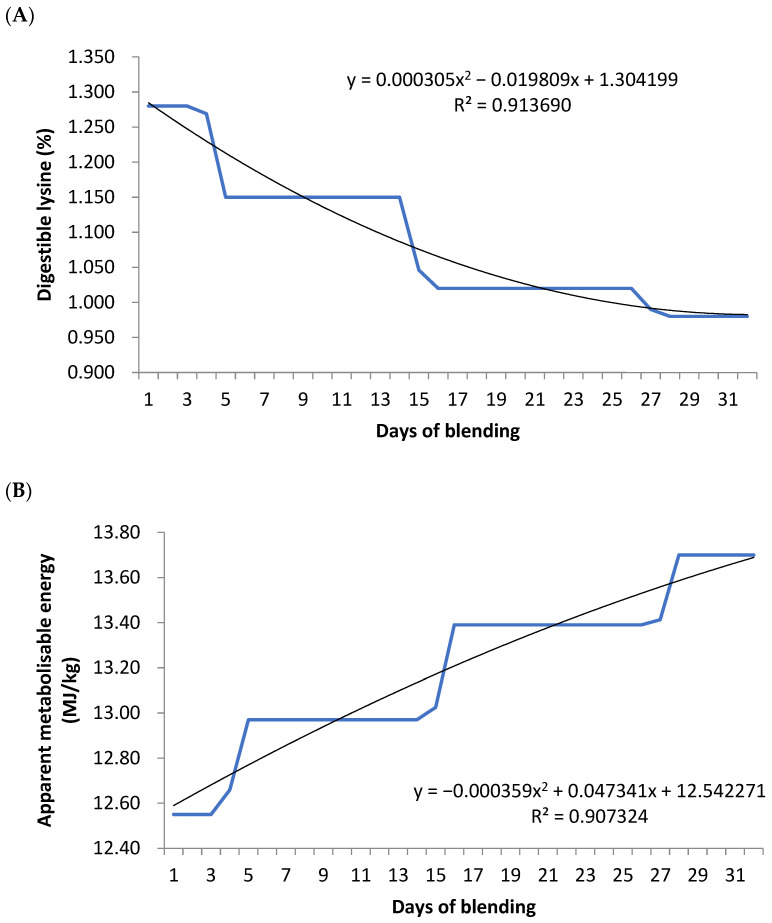
Daily nutrient requirements of standardized ileal digestible lysine (**A**) and apparent metabolisable energy (**B**) of the broiler, as modeled via EFG Software (2019, Version 5.1) Broiler Growth Model growth curves.

The daily nutrient requirements of birds were modeled via EFG Model (2019, Version 5.1; EFG Software, Stellenbosch, South Africa) Broiler Growth Model growth curves (Figure 1) based on Ross 308 (Aviagen, Goulburn, NSW, Australia) performance and nutrient requirement handbooks [11]. From this information, as described above, a protein-dense concentrate and an energy-dense concentrate were blended, and a linear reduction in the protein concentrate for the energy concentrate was calculated. Birds on the precision nutrition adjusted treatment had the diet blends adjusted based on body weight on a weekly basis, where they were moved forward on the feeding schedule to match the requirement of their current weight (not age). Feed Logic (Feedworks Pty Ltd., Romsey, VIC, Australia) feed blending technology was used to accurately mix and deliver the components on a daily basis. Diets were provided as crumbles for starter (days 0–10), and pellets for grower (days 11–21), finisher (days 22–35) and withdrawal (days > 35) phases.

### 2.2. Data Collection

Birds and feed were weighed weekly starting on day 11 to calculate weekly weight gain and feed intake, from which the feed conversion ratio (FCR) was calculated, corrected for mortality by calculating the proportional feed intake per day of the bird that died and removing it from the total for the pen. A total of four birds per pen were moved to metabolic cages (0.44 m^2^ with the space taken up by the feeder removed) on day 21 in a different room but within the same research facility for total excreta collection. The temperature and lighting within the room containing metabolic cages was controlled and followed the same schedule as the main experimental facility. A four-day adaptation period was allowed, and feed intake and excreta output were measured from days 25 to 27 to calculate apparent metabolizable energy (AME). During the period from days 21 to 27, each treatment continued on their daily blends. Thus, due to the blending, there were differences at the point of excreta collection for AME, where the AME of each diet was control: AME = 13.27 MJ and crude protein (CP) = 20.8%; precision nutrition blend: AME = 13.30 MJ and CP = 21.0%; precision nutrition adjusted: AME = 13.33 MJ and CP = 20.8%; and blended standard: AME = 13.32 MJ and CP = 20.7%. On day 28, birds in the metabolic cages were euthanized via electrical stunning (MEFE CAT 44N, Mitchell Engineering Food Equipment, Clontarf, QLD, Australia) followed by cervical dislocation to measure fat pad weights and collect digesta for nutrient digestibility analysis. The jejunum was defined by the end of the duodenal loop through to Meckel’s diverticulum. The ileum was defined by Meckel’s diverticulum through to the ileo-cecal junction. Digesta was collected from the second half of the jejunal and ileal segments, pooled per cage, homogenized and freeze dried (Christ Alpha 1-4 LD plus, Osterode am Harz, Germany). On day 42, four birds per pen were euthanized via electrical stunning followed by cervical dislocation and sampled to measure fat pad, breast, and thigh and drumstick weights. The sex of the birds was also determined and the sex of any remaining birds was determined via their phenotypic characteristics, which were pronounced at this age. The feed cost per kilo of body weight gain was calculated based on both 2020 and 2022 costings by dividing the total cost of diet consumed per pen by the total kilos weigh gain generated per pen. The trial was completed in 2020 and thus the calculated returns were based on this period.

### 2.3. Laboratory Analysis

Nutritional parameters including dry matter (DM), digestible lysine, crude protein (CP), apparent metabolizable energy (AME), calcium (Ca), phosphorus (P) and sodium (Na) in wheat, soybean meal and canola seed were analyzed using near-infrared reflectance spectroscopy (Foss NIR 6500, Hillerød, Denmark), which is standardized with Evonik AMINONIR Advanced calibration. This information was then used to formulate the experimental diets. Excreta samples were dried for 24 h at 80 °C in an air-forced oven. The gross energy (GE) of diets and excreta were determined via bomb calorimetry using an adiabatic calorimeter (Parr 1281 bomb calorimeter, Parr Instruments Co., Moline, IL, USA). The AME (MJ/kg) was calculated by the following equation.

(1) $$AME\;diet=\frac{\left(Feed\;intake\times GE\;diet)-(Excreta\;output\times GE\;excreta\right)}{Feed\;intake}$$

N-corrected (nitrogen-corrected) AME values were calculated by correcting to zero N retention, using the factor of 36.54 kJ/g. N retention was calculated by the following equation:

(2) $$N\;retention\left(\%\right)=\frac{\left(Feed\;intake\times N\;diet\right)-\left(Excreta\;output\times N\;excreta\right)}{\left(Feed\;intake\times N\;diet\right)}\times 100$$

Concentrations of starch in diets and ileal digesta samples were determined by methods described in Mahasukhonthachat et al. [12]. Nitrogen concentrations were determined as outlined in Siriwan et al. [13].

Diets and digesta samples were analyzed for TiO_2_ concentrations in quadruplicate and duplicate, respectively, by the method described by Short et al. [14]. Then the nutrient digestibility (%) was calculated by the following equation.

(3) $$Nutrient\;digestibility\left(\%\right)=\frac{\left(\frac{Nutrient}{Marker}\right)diet-\left(\frac{Nutrient}{Marker}\right)digesta}{\left(\frac{Nutrient}{Marker}\right)diet}\times 100$$

Toe bone samples were collected from sample birds by severing the middle toe through the joint between the 2nd and 3rd tarsal bones from the distal end on days 28 and 42 per cage. Toes from each cage were pooled and the composite samples dried to a constant weight at 100 °C and then ashed in a muffle furnace at 550 °C for 16 h for the assessment of bone mineralisation as described by Potter [15].

### 2.4. Statistical Analysis

R Commander (version 3.3.1, R Foundation for Statistical Computing, Vienna, Austria) was used to analyze data. Data were tested for normality and variance homogeneity and analyzed via one-way ANOVA to test statistical differences between the treatments.

The model used may be represented as

$$y_{ij}=\mu +\tau_{i}+\varepsilon_{ij}$$

where *y_ij_* represents the *j*-th observation on the *i*-th treatment, *µ* represents the experimental effect, $\tau_{i}\;$represents the *i*-th treatment effect, and $\varepsilon_{ij}$ represents the random error present in the *j*-th observation on the *i*-th treatment.

Tukey’s post hoc test was used to identify pairwise differences between the treatment means from significant ANOVA results. The *p*-value ≤ 0.05 was considered significant.

## 3. Results

### 3.1. Growth Performance

The effects of dietary treatments on weight gain, feed intake and FCR are presented in Table 6, Table 7 and Table 8, respectively. The dietary treatments did not affect weight gain and feed intake over the entire study (Table 6 and Table 7). From days 14 to 21, birds offered the precision nutrition diets and blended standard diets had a lower FCR compared to those fed the control diet (*p* < 0.001, Table 8). Over the study there was a difference in bird weight between the treatments. Thus, as heavier birds have a higher FCR, weight corrected FCR was calculated for the study duration (11 to 42 days) where a correction of 3 points of FCR was applied to each pen for treatments two to four for every 100 g of weight gain greater than the average of the control treatment. There was a significant difference in weight corrected FCR, where the precision nutrition adjusted treatment significantly improved the corrected FCR compare to the control and blended standard diets treatments (*p* = 0.041). Mortality and culls over the trial totaled 5.6% and was unrelated to dietary treatment.

### 3.2. Body Weight Uniformity and Feed Cost

The effects of precision nutrition on feed cost and body weight uniformity from day 11 to 42 are given in Table 9. The blended standard diet reduced the body weight coefficient of variation at days 28 and 35 compared to the control. Both precision nutrition treatment groups recorded highest body weights compared to the control and blended standard diet on day 42 (Table 10). Feed costs did not differ significantly between treatments, but precision nutrition adjusted treatments saved 3.2 cents/kg body weight, or 4.13 percent feed cost.

### 3.3. Carcass Yield

Relative weights of fat pad, breast, thigh and drumstick are shown in Table 11. Relative fat pad, breast, thigh and drumstick weight on day 28 and 42 was not affected by dietary treatments.

### 3.4. Apparent Ileal Nutrient Digestibility

The effects of dietary treatments on apparent ileal digestibilities (%) of dry matter, protein and starch on day 28 are shown in Table 12. Feeding the blended standard diet reduced apparent ileal starch digestibility compared to the control and precision nutrition diets on day 28 (*p* = 0.037). The apparent ileal digestibility of dry matter on day 28 was not significantly affected in precision nutrition diets compared to the control and blended standard diets (*p* > 0.05).

### 3.5. Nutrient Utilization and Excreta Moisture

The effects of dietary treatments on AME, N-corrected AME, and excreta moisture from days 25 to 27 are shown in Table 13. Both precision nutrition blended and adjusted treatments had the greatest AME (*p* = 0.002) and N-corrected AME (*p* = 0.013) from days 25 to 27 among the dietary treatments. The blended standard diet led to the lowest energy utilization from days 25 to 27, similar to the control group. Excreta moisture content was not influenced by the dietary treatments from days 25 to 27.

### 3.6. Toe Ash

The effects of dietary treatments on toe ash at days 28 and 42 are shown in Table 14. There was no significant influence of dietary treatments on toe ash at both day 28 and day 42.

## 4. Discussion

Feed accounts for more than 65% of the total cost of chicken-meat production [16]. Hence, increasing feed efficiency could increase the productivity of the poultry industry and improve economic sustainability. The present study demonstrated that precision nutrition diet programs may improve feed efficiency in the early stage of broiler production compared to the conventional phase-feeding regime, as evidenced by an improved FCR between days 14 and 21 post-hatch. It is interesting to note that the impact of the precision nutrition program on FCR was observed over the first half of the study. The grower and finisher period immediately follow the greatest change in protein concentration during the diet change (starter to grower diets and grower to finisher diets, respectively); therefore, the greatest response should be seen immediately following these periods. The daily nutrient requirement in the present study was calculated by the EFG broiler growth model. Instructively, in a previous study, Gutierrez et al. [17] also found that blending two dietary components to meet the daily nutrient requirement as calculated by the EFG Broiler Growth Model improved weight gain and feed efficiency, especially from days 21 to 28 and 35 to 42, thereby reducing feed costs per kilogram of weight gain.

Gutierrez et al. [17] study fed the precision nutrition diets based on the calculated nutrient requirement from the EFG Model, but did not adjust the blends based on the birds’ actual performance. In the present study, birds offered the precision nutrition treatments grew heavier than other treatments as the experiment progressed. As heavier birds have a higher FCR, weight corrected FCR was calculated for the study duration (11 to 42 days), and it was found that birds offered the adjusted precision nutrition treatment improved weight corrected FCR by 7.8% compared to the control and blended standard diet treatments. Additionally, the precision nutrition adjusted treatment finished with birds reaching the final target blend (100% low protein, high energy concentrate) five days sooner than the precision nutrition treatment. This resulted in a reduction in feed cost of 3.2 cents/kg body weight, or by 4.13% compared to the control diet (based on 2020 costings). Thus, the present study is consistent with the Gutierrez et al. [17] findings.

Feed intake and growth rate are associated with greater body fat accumulation in broiler chickens [18]. Considering the low economic value of broiler fat, and consumer preferences for lean meat, excessive deposition of fat presents a challenge for poultry producers and consumers alike. Birds on precision nutrition feeding programs may accumulate less fat within the body due to a reduced dietary over-supply of energy which can become stored as fat [18]. However, in this study, birds receiving precision nutrition treatments showed no significant changes to fat pads and additionally, no significant difference in breast, thigh and drumstick weight at 28 and 42 days of age. In a similar study, Roush et al. [19] showed that broilers fed blended diets (starter and grower, starter and finisher, and grower and finisher), similar to that of the present study’s ‘blended standard diets’ treatment, did not differ significantly in final body weight, fat pads, or breast muscle from those offered traditional 4 phase feeding programs. Entire fat pad removal from carcasses can be difficult, and thus it may be worthwhile exploring if birds offered high-protein low energy diets may exhibit reduced mRNA expression of hepatic malic enzyme (HME), acetyl coenzyme carboxylase (ACC), and fatty acid synthase (FAS); key enzymes in the de novo lipogenesis pathway in chickens [20], however this has not yet been explored. Contrary to the present study and the Roush et al. [19] study, Moss et al. [21] found that carcass dressed weight increased from 2.282 to 2.502 kg (*p* = 0.001), resulting in a decrease in the cost per kilogram of chicken-meat from 71.4 cents to 66.3 cents under a precision feeding program in comparison to a standard 4 phase feeding regimen at 42 days. Thus, the effect of precision nutrition programs on carcass composition is somewhat conflicting.

Improved flock uniformity may bring savings at the processing plant and is also a potential welfare indicator, associated with increased rejection rates at slaughter [22]. It is possible that reducing excessive nutrient supply may have contributed to the observed reduction in CV (improved flock uniformity) at 14 and 42 days within the precision nutrition adjusted treatment compared to the control treatment in the current study. The number of precision feeding studies on broilers is lacking, however the effect of precision feeding for broiler breeders has gained significant recent attention. Zuidhof [23] recorded that in comparison to conventional feeding in broiler breeders, the precision feeding station developed by his laboratory has produced 100% flock uniformity for seven of the last 10 weeks of a pilot study. It was explained in Zuidhof [23] that the precise feeding stations (automatic weighing and tracking) can provide real-time adjustments of nutrients, preventing aggressive birds from overeating and providing equal feeding opportunities to all birds. Nevertheless, this approach is substantially different from the precision nutrition programs discussed in the present study, as it manipulates feed intake (i.e., precision feeding) and not the nutrient content of the diet (i.e., precision nutrition).

In addition to the potential improvements in efficiency and CV, precision nutrition programs also present some logistical benefits for chick performance. The poultry industry faces a logistical challenge when it starts a new batch of broilers, of how to dispose of unused feed. While it can be removed, this provides an extra cost and wastage [24]. Thus, the withdrawal feed remaining in the silo unavoidably gets fed to the next batch of young chicks that arrive to the farm until it runs out and is replaced with the new starter feed. Precision nutrition programs avoid this logistics issue, as the concentrates are required for all stages, and so the blend percentages only need to be reset for the next flock. Thus, any leftover feed can be utilized while still meeting the next flock’s nutrient requirements.

The content of soybean meal was higher in the low protein blend than the finisher and withdrawal diets, and the content of oil was higher in the high-protein blend than the starter diet in the present study. Thus, precision nutrition diets are sensitive to the price of soybean meal and the price of oil compared to the control diet in the present study. To demonstrate this sensitivity, diet cost calculations are provided for both 2020 and updated for 2022 which saw an increased soybean meal and oil price over this period due to significant global events [25]. However, this may be avoided partly by utilizing alternative protein and oil sources that are cheaper.

The blended standard diet treatment of the present study was included to determine if some of the benefits of blending diets could be achieved by gradually blending the four feeding phases, which may aid the adoption of the feeding strategy. We hypothesized that while not precisely meeting protein and energy requirements as well as the protein and energy blends, it may still generate benefit from eliminating sudden diet changes. It has been demonstrated in multiple animal species that a sudden diet change disrupts the gut via the microbiome [26,27,28]; thus, a gradual blending of diets would provide less disruption to the gut. While we did not measure gut microbiome in the present study, this effect appears not to have been realized, as blended standard diets saw a reduction in multiple parameters including nutrient digestibility and energy utilization. Thus, blending the standard 4-phase diets may not present an alternative to the concentrate blends in the precision nutrition diets.

The apparent metabolizable energy (AME) adjusted for zero nitrogen retention (AMEn) is commonly used to evaluate the energy value of ingredients [29]. Precision nutrition adjusted treatments showed the highest AME among dietary treatments from 25 to 27 days of age, which was significantly improved in comparison to the control. The AME was measured following the feed swap from grower to finisher diets for the control treatment. Thus, the improvement may have been attributed to either (i) more precisely meeting the energy requirement, and/or (ii) the lack of disturbance in the gut from a sudden diet change. While there is very limited research on the impact of changing feeding phases on the gut and performance of broiler chickens, there is a proven link between the gut microbiome and the maintenance of host circadian rhythms and metabolic homeostasis in several species [30,31]. Therefore, if the gut microbiome is disturbed under a sudden feed change, it is sensible that metabolic homeostasis would be disrupted which would disrupt the performance of the chicken [32]. Thus, this may be another avenue by which precision nutrition may enhance broiler performance. However, as we did not see any effect from the blended standard diets treatment, blending diets to reduce impact on the gut microbiome may not be the main reason for the improvements seen.

The present study demonstrates the benefits of precision feeding regimes. However, there are some barriers to practical industry adoption. Firstly, farms usually only have one silo per shed, and so investment would need to be made in both a feed blending system and an extra silo. However, as estimated by Moss et al. [5], the cost of the initial outlay for equipment would be recovered within a short timeframe. Secondly, while the two silos of energy concentrates do create logistical advantages as discussed above, this system is more complex and would require farm staff who are interested in learning and utilizing this technology. There does appear to be an appetite of producers for such technology, as a recent survey revealed that while broiler producers were generally unfamiliar with what technology is available in precision livestock farming for broiler production systems, they would be willing to adopt new technology given it proved to increase farm productivity and profitability [33].

## 5. Conclusions

It is concluded that precision nutrition may improve broiler weight corrected FCR. Improvements in energy utilization and flock uniformity were also demonstrated in birds offered precision nutrition programs in the present study.

## Figures and Tables

**Table 1:** Schedule of dietary treatments.

Treatment	Description
1	Control (four phases)
2	Precision nutrition blend (daily blended protein and energy concentrate based on trial day)
3	Precision nutrition adjusted (daily blended protein and energy concentrate based on actual weekly bird weight)
4	Blended standard diets (control four diet phases gradually blended)

**Table 2:** Formulation of experimental diets/concentrates (%).

Ingredient	Cost (AUD)/Tonne	Starter	Grower	Finisher	Withdrawal	High-Protein/Low-Energy	Low-Protein/High-Energy
Soybean meal	510	34.0	27.8	21.9	22.3	34.3	22.5
Wheat	290	55.9	59.9	64.1	63.4	55.4	63.3
Canola seed	340	3.0	6.0	8.0	8.0	3.0	8.0
Limestone	115	1.341	1.198	1.062	0.811	1.341	0.811
Salt	245	0.192	0.175	0.177	0.161	0.192	0.161
Monodicalcium phosphate	975	0.843	0.666	0.488	0.289	0.842	0.288
Sodium bicarbonate	345	0.298	0.268	0.266	0.180	0.298	0.180
Vegetable oil	2500	2.695	2.688	2.794	3.781	2.900	3.757
Betaine 38%	1800	0.130	0.130	0.130	0.130	0.130	0.130
L-lysine sulfate	2550	0.369	0.340	0.315	0.221	0.368	0.221
DL-methionine	3600	0.417	0.361	0.317	0.269	0.420	0.270
L-threonine	3650	0.139	0.111	0.085	0.066	0.140	0.067
L-Valine	6700	0.011				0.012	
choline chloride 75% L	450	0.025	0.025	0.020	0.020	0.025	0.020
Vitamin + mineral premix ^1^	8000	0.450	0.250	0.250	0.200	0.450	0.200
Xylanase ^2^	12,000	0.025	0.025	0.025	0.025	0.025	0.025
Phytase ^3^	12,000	0.030	0.030	0.030	0.030	0.030	0.030
Diet cost (AUD)/tonne		499.63	467.08	453.54	466.84	505.01	467.03

^1^ Vitamin and mineral premix per kg diet: vitamin A, 12 million international units (MIU); vitamin D, 5 MIU; vitamin E, 75 mg; vitamin K, 3 mg; nicotinic acid, 55 mg; pantothenic acid, 13 mg; folic acid, 2 mg; riboflavin, 8 mg; cyanocobalamin, 0.016 mg; biotin, 0.25 mg; pyridoxine, 5 mg; thiamine, 3 mg; antioxidant, 50 mg; Cu, 16 mg as copper sulfate; Mn, 60 mg as manganese sulfate; Mn, 60 mg as manganous oxide; I, 0.125 mg as potassium iodide; Se, 0.3 mg; Fe, 40 mg, as iron sulfate; Zn, 50 mg as zinc oxide; Zn, 50 mg as zinc sulfate. ^2^ Xylanase 8000 L, Danisco Animal Nutrition (IFF), Oegstgeest, The Netherlands. ^3^ Axtra PHY 5000 L, Danisco Animal Nutrition (IFF), Oegstgeest, The Netherlands.

**Table 3:** Calculated and analyzed nutrient composition of experimental diets/concentrates (%, unless otherwise stated).

Nutrient	Starter	Grower	Finisher	Withdrawal	High-Protein/Low-Energy	Low-Protein/High-Energy
Calculated						
Dry matter	90.56	90.54	90.54	90.55	90.579	90.552
AMEn, MJ/kg ^1^	12.55	12.97	13.39	13.70	12.589	13.690
Crude protein	23.43	21.58	19.70	19.73	23.508	19.772
SID Lysine ^2^	1.280	1.150	1.020	0.980	1.285	0.983
SID Methionine ^2^	0.707	0.634	0.572	0.526	0.711	0.528
SID Methionine + cysteine ^2^	0.950	0.870	0.800	0.755	0.954	0.757
SID Threonine ^2^	0.860	0.770	0.680	0.666	0.863	0.668
SID Tryptophan ^2^	0.276	0.254	0.231	0.233	0.277	0.233
SID Isoleucine ^2^	0.860	0.780	0.700	0.706	0.863	0.708
SID Leucine ^2^	1.487	1.358	1.227	1.236	1.493	1.240
SID Valine ^2^	0.960	0.873	0.795	0.801	0.964	0.803
SID Arginine ^2^	1.397	1.256	1.114	1.124	1.404	1.128
Ca	0.960	0.870	0.780	0.647	0.960	0.647
Non-phytate P	0.480	0.435	0.390	0.350	0.480	0.350
Crude fiber	2.691	2.750	2.767	2.766	2.689	2.768
Sodium	0.195	0.180	0.180	0.150	0.195	0.150
Chloride	0.200	0.190	0.190	0.180	0.200	0.180
Potassium	0.992	0.902	0.814	0.820	0.995	0.822
Crude fat	5.507	6.574	7.394	8.366	5.705	8.343
Analyzed						
Dry matter	88.1	86.7	87.0	87.3	87.6	87.9
Gross energy, MJ/kg	17.15	17.08	17.38	17.66	17.02	17.84
Protein (N × 6.25)	25.0	23.0	19.5	19.7	24.8	19.3
Starch	33.5	34.0	38.0	38.7	31.7	39.4

^1^ AMEn calculated from the AMEn of ingredients, which was estimated from Near-Infra Red spectroscopy (Foss NIR 6500, Hillerød, Denmark) calculation based on the WPSA equations [10]. ^2^ Digestible basis; standardized ileal digestibility (SID). Digestible amino acid coefficients for raw ingredients were determined by Near-Infra Red spectroscopy (Foss NIR 6500, Hillerød, Denmark) standardized with Evonik AMINONIR^®^ Advanced calibration (Evonik, Essen, Germany).

**Table 4:** Record of dietary blends offered from days 11 to 42 post-hatch.

Day	Control	Precision Nutrition Blend				Precision Nutrition Adjusted				Blended Standard			
		Blend 1	%	Blend 2	%	Blend 1	%	Blend 2	%	Blend 1	%	Blend 2	%
11	Starter	Hi Pro ^1^	100	Lo Pro ^2^	0	Hi Pro	100	Lo Pro	0	Starter	100	Grower	0
12	Starter	Hi Pro	94	Lo Pro	6	Hi Pro	94	Lo Pro	6	Starter	66	Grower	34
13	Starter	Hi Pro	88	Lo Pro	12	Hi Pro	88	Lo Pro	12	Starter	33	Grower	67
14	Grower	Hi Pro	82	Lo Pro	18	Hi Pro	82	Lo Pro	18	Grower	100	Finisher	0
15	Grower	Hi Pro	76	Lo Pro	24	Hi Pro	71	Lo Pro	29	Grower	91	Finisher	9
16	Grower	Hi Pro	71	Lo Pro	29	Hi Pro	65	Lo Pro	35	Grower	82	Finisher	18
17	Grower	Hi Pro	65	Lo Pro	35	Hi Pro	60	Lo Pro	40	Grower	73	Finisher	27
18	Grower	Hi Pro	60	Lo Pro	40	Hi Pro	56	Lo Pro	44	Grower	64	Finisher	36
19	Grower	Hi Pro	56	Lo Pro	44	Hi Pro	51	Lo Pro	49	Grower	55	Finisher	45
20	Grower	Hi Pro	51	Lo Pro	49	Hi Pro	47	Lo Pro	53	Grower	46	Finisher	54
21	Grower	Hi Pro	47	Lo Pro	53	Hi Pro	42	Lo Pro	58	Grower	37	Finisher	63
22	Grower	Hi Pro	42	Lo Pro	58	Hi Pro	38	Lo Pro	62	Grower	28	Finisher	72
23	Grower	Hi Pro	38	Lo Pro	62	Hi Pro	34	Lo Pro	66	Grower	19	Finisher	81
24	Grower	Hi Pro	34	Lo Pro	66	Hi Pro	31	Lo Pro	69	Grower	10	Finisher	90
25	Finisher	Hi Pro	31	Lo Pro	69	Hi Pro	27	Lo Pro	73	Finisher	100	WD ^3^	0
26	Finisher	Hi Pro	27	Lo Pro	73	Hi Pro	24	Lo Pro	76	Finisher	92.3	WD	7.7
27	Finisher	Hi Pro	24	Lo Pro	76	Hi Pro	21	Lo Pro	79	Finisher	84.6	WD	15.4
28	Finisher	Hi Pro	21	Lo Pro	79	Hi Pro	18	Lo Pro	82	Finisher	76.9	WD	23.1
29	Finisher	Hi Pro	18	Lo Pro	82	Hi Pro	13	Lo Pro	87	Finisher	69.2	WD	30.8
30	Finisher	Hi Pro	16	Lo Pro	84	Hi Pro	11	Lo Pro	89	Finisher	61.5	WD	38.5
31	Finisher	Hi Pro	13	Lo Pro	87	Hi Pro	9	Lo Pro	91	Finisher	53.8	WD	46.2
32	Finisher	Hi Pro	11	Lo Pro	89	Hi Pro	7	Lo Pro	93	Finisher	46.1	WD	53.9
33	Finisher	Hi Pro	9	Lo Pro	91	Hi Pro	6	Lo Pro	94	Finisher	38.4	WD	61.6
34	Finisher	Hi Pro	7	Lo Pro	93	Hi Pro	4	Lo Pro	96	Finisher	30.7	WD	69.3
35	Finisher	Hi Pro	6	Lo Pro	94	Hi Pro	3	Lo Pro	97	Finisher	23	WD	77
36	Finisher	Hi Pro	4	Lo Pro	96	Hi Pro	0	Lo Pro	100	Finisher	15.3	WD	84.7
37	Finisher	Hi Pro	3	Lo Pro	97	Hi Pro	0	Lo Pro	100	Finisher	7.6	WD	92.4
38	Withdrawal	Hi Pro	2	Lo Pro	98	Hi Pro	0	Lo Pro	100	WD	100	WD	0
39	Withdrawal	Hi Pro	1	Lo Pro	99	Hi Pro	0	Lo Pro	100	WD	100	WD	0
40	Withdrawal	Hi Pro	1	Lo Pro	99	Hi Pro	0	Lo Pro	100	WD	100	WD	0
41	Withdrawal	Hi Pro	0	Lo Pro	100	Hi Pro	0	Lo Pro	100	WD	100	WD	0
42	Withdrawal	Hi Pro	0	Lo Pro	100	Hi Pro	0	Lo Pro	100	WD	100	WD	0

^1^ Hi Pro, high-protein and low-energy concentrate; ^2^ Lo Pro, low-protein and high-energy concentrate; ^3^ WD, withdrawal.

**Table 5:** Daily calculated intake of standardized ileal digestible lysine (SIDL%) and AME (MJ/kg) of each treatment based on average treatment intake.

Day	Control		Precision Nutrition Blend		Precision Nutrition Adjusted		Blended Standard	
	SIDL (%)	AME (MJ/Kg)	SIDL (%)	AME (MJ/Kg)	SIDL (%)	AME (MJ/Kg)	SIDL (%)	AME (MJ/Kg)
11	0.060	0.59	0.060	0.58	0.059	0.58	0.058	0.57
12	0.068	0.67	0.067	0.67	0.066	0.66	0.064	0.65
13	0.076	0.74	0.073	0.75	0.073	0.74	0.069	0.74
14	0.081	0.80	0.077	0.81	0.077	0.80	0.071	0.80
15	0.091	1.03	0.095	1.00	0.096	1.03	0.093	1.07
16	0.099	1.12	0.102	1.10	0.103	1.13	0.101	1.17
17	0.108	1.21	0.109	1.19	0.110	1.23	0.108	1.27
18	0.116	1.31	0.116	1.29	0.118	1.33	0.115	1.37
19	0.125	1.42	0.124	1.40	0.126	1.45	0.124	1.49
20	0.135	1.52	0.132	1.52	0.134	1.57	0.132	1.61
21	0.143	1.62	0.138	1.62	0.140	1.67	0.138	1.71
22	0.137	1.54	0.145	1.72	0.134	1.62	0.139	1.75
23	0.145	1.64	0.152	1.84	0.141	1.73	0.146	1.86
24	0.153	1.72	0.158	1.94	0.147	1.82	0.152	1.96
25	0.136	1.79	0.158	1.96	0.147	1.84	0.151	1.99
26	0.142	1.86	0.163	2.04	0.151	1.92	0.157	2.07
27	0.146	1.92	0.166	2.11	0.154	1.98	0.161	2.14
28	0.150	1.97	0.169	2.18	0.158	2.05	0.165	2.20
29	0.164	2.16	0.173	2.25	0.162	2.15	0.158	2.12
30	0.168	2.20	0.175	2.30	0.164	2.20	0.161	2.17
31	0.172	2.26	0.179	2.37	0.168	2.26	0.165	2.23
32	0.176	2.31	0.181	2.42	0.170	2.31	0.168	2.28
33	0.181	2.38	0.186	2.50	0.175	2.38	0.173	2.36
34	0.186	2.44	0.189	2.57	0.179	2.45	0.177	2.42
35	0.189	2.49	0.192	2.62	0.181	2.50	0.179	2.47
36	0.173	2.27	0.185	2.53	0.180	2.51	0.170	2.35
37	0.177	2.32	0.188	2.59	0.185	2.57	0.173	2.41
38	0.170	2.38	0.188	2.60	0.185	2.57	0.172	2.41
39	0.173	2.42	0.191	2.64	0.188	2.62	0.175	2.40
40	0.177	2.47	0.195	2.70	0.192	2.68	0.179	2.45
41	0.181	2.53	0.199	2.76	0.196	2.74	0.183	2.50
42	0.185	2.58	0.203	2.82	0.201	2.79	0.187	2.56
Total intake	4.585	57.68	4.825	61.38	4.659	59.90	4.567	59.55

**Table 6:** Effects of dietary treatments on weekly and total (d11 to d42 post-hatch) weight gain (g/bird).

Treatment	Period (Days Post-Hatch)							
	11 to 14	14 to 21	21 to 28	28 to 35	35 to 42	11 to 28	28 to 42	11 to 42
Control (4 phases)	197	543	718	741	655	1445	1396	2841
Precision nutrition blend	195	568	771	775	710	1534	1486	3020
Precision nutrition adjusted	196	569	742	783	751	1509	1541	3041
Blended standard diets	194	568	724	744	671	1486	1414	2900
SEM ^1^	2.43	13.61	21.87	23.28	43.48	30.78	49.71	57.27
*p* value	0.781	0.516	0.353	0.478	0.417	0.228	0.184	0.054

^1^ SEM, standard error of the mean.

**Table 7:** Effects of dietary treatments on weekly and total (d11 to d42 post-hatch) feed intake (g/bird).

Treatment	Period (Days Post-Hatch)							
	11 to 14	14 to 21	21 to 28	28 to 35	35 to 42	11 to 28	28 to 42	11 to 42
Control (4 phases)	223	711	941	1213	1247	1902	2459	4361
Precision nutrition blend	221	700	1033	1254	1364	1995	2617	4612
Precision nutrition adjusted	219	720	969	1193	1350	1944	2543	4451
Blended standard diets	216	738	1044	1184	1264	1999	2447	4446
SEM ^1^	2.54	22.93	42.44	30.65	43.06	52.97	57.32	93.59
*p* value	0.288	0.693	0.277	0.413	0.146	0.536	0.114	0.306

^1^ SEM, standard error of the mean.

**Table 8:** Effects of treatments on weekly, total (d11 to d42 post-hatch) and weight corrected total feed conversion ratio (g/g).

Treatment	Period (Days Post-Hatch)								Corrected FCR 11 to 42d
	11 to 14	14 to 21	21 to 28	28 to 35	35 to 42	11 to 28	28 to 42	11 to 42	
Control (4 phases)	1.132	1.397 ^a^	1.409	1.623	1.803	1.320	1.791	1.539	1.539 ^b^
Precision nutrition blend	1.132	1.203 ^b^	1.331	1.578	1.847	1.273	1.768	1.528	1.489 ^ab^
Precision nutrition adjusted	1.114	1.207 ^b^	1.359	1.526	1.766	1.289	1.659	1.464	1.419 ^a^
Blended standard diets	1.117	1.255 ^b^	1.357	1.630	1.812	1.318	1.749	1.535	1.526 ^b^
SEM ^1^	0.010	0.019	0.034	0.028	0.060	0.019	0.058	0.026	0.032
*p* value	0.480	<0.001	0.358	0.058	0.845	0.292	0.407	0.161	0.041

^ab^ Means within columns not sharing a common superscript are significantly different at the 5% level of probability. ^1^ SEM, standard error of the mean.

**Table 9:** Effect of treatments on coefficient of variation (CV; of the individual weights of birds within a pen) at days 14, 21, 28, 35 and 42 post-hatch, and feed cost (AUD) per kilo body weight at d42.

Treatment	14 Days	21 Days	28 Days	35 Days	42 Days	Feed Cost (AUD)/kg Body Weight (2020 Prices)	Feed Cost (AUD)/kg Body Weight (2022 Prices)
Control	8.85 ^a^	10.03	12.08 ^a^	14.02 ^a^	15.24 ^a^	0.774	0.935
Precision nutrition blend	8.96 ^a^	8.94	8.83 ^b^	9.19 ^b^	11.04 ^b^	0.771	0.920
Precision nutrition adjusted	6.71 ^b^	7.14	10.55 ^ab^	10.02 ^b^	9.26 ^b^	0.742	0.909
Blended standard diets	9.64 ^a^	8.37	8.86 ^b^	10.52 ^b^	12.29 ^ab^	0.771	0.930
SEM ^1^	0.569	0.96	0.67	1.11	1.25	0.011	0.014
*p* value	0.012	0.222	0.006	0.026	0.019	0.209	0.581

^ab^ Means within columns not sharing a common superscript are significantly different at the 5% level of probability. ^1^ SEM, standard error of the mean.

**Table 10:** Effects of dietary treatments on body weight (g) (d11 to d42 post-hatch).

Treatment	D11	D14	D21	D28	D35	D42
Control (4 phases)	356	553	1094	1801	2542	3197 ^a^
Precision nutrition blend	360	556	1124	1895	2670	3381 ^b^
Precision nutrition adjusted	365	561	1130	1872	2655	3428 ^b^
Blended standard diets	361	554	1122	1846	2590	3315 ^a^
SEM ^1^	1.16	1.12	5.06	12.75	18.77	31.58
*p* value	0.256	0.641	0.419	0.202	0.135	0.044

^ab^ Means within columns not sharing a common superscript are significantly different at the 5% level of probability. ^1^ SEM, standard error of the mean.

**Table 11:** Effects of dietary treatments on carcass parameters including relative fat pad weight (g/kg) at 28 and 42 days post-hatch, relative breast (g/kg), thigh (g/kg) and drumstick weights (g/kg) at 42 days post-hatch.

Treatment	D28 Fat Pad Weight	D42 Fat Pad Weight	D42 Breast Weight	D42 Thigh Weight	D42 Drumstick Weight
Control (4 phases)	7.29	10.63	100.75	51.1	43.22
Precision nutrition blend	7.06	9.80	98.95	51.2	42.52
Precision nutrition adjusted	6.96	8.70	96.40	50.6	42.19
Blended standard diets	8.04	10.46	97.60	51.1	43.81
SEM ^1^	0.381	0.513	2.612	0.906	0.761
*p* value	0.188	0.055	0.598	0.900	0.320

^1^ SEM, standard error of the mean.

**Table 12:** Effects of dietary treatments on apparent dry matter, protein (N) and starch ileal digestibility as a percentage at 28 days post-hatch.

Treatment	Apparent Ileal Dry Matter Digestibility (%)	Apparent Ileal Protein Digestibility (%)	Apparent Ileal Starch Digestibility (%)
Control (4 phases)	67.96	79.45	95.65 ^a^
Precision nutrition blend	67.35	79.12	95.87 ^a^
Precision nutrition adjusted	68.49	80.58	95.70 ^a^
Blended standard diets	65.58	78.01	91.99 ^b^
SEM ^1^	0.767	0.818	1.024
*p* value	0.081	0.234	0.037

^ab^ Means within columns not sharing a common superscript are significantly different at the 5% level of probability. ^1^ SEM, standard error of the mean.

**Table 13:** Effects of dietary treatments on apparent metabolisable energy (AME; MJ/kg DM), N corrected AME (AMEn; MJ/kg DM) and excreta moisture (%) from 25 to 27 days post-hatch.

Treatment	AME	AMEn	Excreta Moisture
Control (4 phases)	12.34 ^ab^	11.62 ^ab^	79.9
Precision nutrition blend	12.56 ^bc^	11.75 ^bc^	80.9
Precision nutrition adjusted	12.62 ^c^	11.78 ^bc^	81.6
Blended standard diets	12.16 ^a^	11.40 ^a^	79.3
SEM ^1^	0.080	0.081	0.777
*p* value	0.002	0.013	0.162

^abc^ Means within columns not sharing a common superscript are significantly different at the 5% level of probability. ^1^ SEM, standard error of the mean.

**Table 14:** Effects of dietary treatments on toe ash (%) at 28 and 42 days post-hatch.

Treatment	Day 28	Day 42
Control (4 phases)	11.12	10.98
Precision nutrition blend	11.14	10.64
Precision nutrition adjusted	11.57	10.94
Blended standard diets	10.87	10.81
SEM ^1^	0.301	0.357
*p* value	0.437	0.910

^1^ SEM, standard error of the mean.

## Data Availability

The original contributions presented in this study are included in the article. Further inquiries can be directed to the corresponding author.
